# Type II cGMP-dependent protein kinase inhibits epidermal growth factor-induced phosphatidylinositol-3-kinase/Akt signal transduction in gastric cancer cells

**DOI:** 10.3892/ol.2013.1630

**Published:** 2013-10-15

**Authors:** MIN WU, YONGCHANG CHEN, LU JIANG, YUEYING LI, TING LAN, YING WANG, HAI QIAN

**Affiliations:** Department of Physiology, School of Medical Science and Laboratory Medicine, Jiangsu University, Zhenjiang, Jiangsu 212013, P.R. China

**Keywords:** Type II cGMP-dependent protein kinase, phosphatidylinositol-3-kinase/Akt-mediated signal transduction, apoptosis, gastric cancer cells

## Abstract

Our previous study revealed that Type II cGMP-dependent protein kinase (PKG II) inhibits epidermal growth factor (EGF)-induced MAPK/ERK and MAPK/JNK-mediated signal transduction through the inhibition of the phosphorylation/activation of the EGF receptor (EGFR). As EGFR also mediates several other signal transduction pathways besides MAPK-mediated pathways, the present study was designed to investigate whether PKG II was able to inhibit EGF/EGFR-induced phosphatidylinositol-3-kinase (PI3K)/Akt-mediated signal transduction. The AGS human gastric cancer cell line was infected with adenoviral constructs encoding a cDNA of PKG II (Ad-PKG II) to increase the expression of PKG II, and treated with 8-pCPT-cGMP to activate the enzyme. Western blotting was used to detect the phosphorylation/activation of the key components of the signal transduction pathway, including EGFR, PI3K, Akt, mTOR and NF-κB. The levels of apoptosis-related proteins, including Bax, Bcl-2, caspase 9 and DNA fragment factor (DFF), were also determined by western blotting. Terminal deoxynucleotidyl transferase-mediated dUTP nick end-labeling staining was used to detect the apoptosis of the AGS cells. The results revealed that EGF treatment increased the phosphorylation (activation) of EGFR, PI3K, Akt and mTOR, and increased the nuclear localization (activation) of NF-κB. EGF treatment also reduced the apoptosis of the AGS cells and increased the expression of the anti-apoptotic protein, Bcl-2, but had no effect on the expression of the pro-apoptotic protein, Bax, and did not alter the levels of caspase 9 and DFF. Increasing the PKG II activity of AGS cells by infecting them with Ad-PKG II and stimulating them with 8-pCPT-cGMP inhibited the EGF-induced activation of EGFR, PI3K, Akt, mTOR and NF-κB; caused an increase in caspase 9 breakdown (activation) and DFF levels; and reversed the anti-apoptotic effect of EGF. The results suggest that PKG II may also inhibit EGF-induced signal transduction of PI3K/Akt-mediated pathways, and further confirm that PKG II is able to block the activation of EGFR.

## Introduction

The phosphatidylinositol-3-kinase (PI3K)/Akt-mediated signal transduction pathway is closely associated with tumorigenesis and has been used as a target of caner therapy (1). PI3K is able to phosphorylate the third OH group on the inositol ring of phosphatidylinositol (PI), and the main product of the phosphorylation is phosphatidylinositol-3,4,5-triphosphate (PIP3). PIP3 acts as the second messenger to activate downstream components of the signal transduction pathway. Akt, also known as protein kinase B, is a serine/threonine-specific protein kinase that is crucial in the regulation of cellular proliferation, differentiation, apoptosis and migration. PIP3 is able to bind with Akt and cause the translocation of Akt from the plasma to the membrane and activate the enzyme (2,3). PI3K/Akt-mediated signal transduction may be initiated by interacting with receptor tyrosine kinases (RTKs) or by binding with the small G protein, Ras (4). The epidermal growth factor receptor (EGFR) is a key member of the RTK family. When ligands bind with EGFR, autophosphorylation of EGFR occurs and the phosphorylated tyrosine site is able to recruit PI3K to the C-terminal domain of EGFR, causing the phosphorylation/activation of PI3K, initiating the associated signal transduction (5).

cGMP-dependent protein kinases (PKGs) are serine/threonine kinases and, currently, two types of PKGs have been identified in mammalian cells, Type I (PKG I) and Type II (PKG II) (6,7). PKG I has been shown to suppress the growth of tumor cells and has been identified as a tumor suppressor (8). However, the antitumor effect of PKG II has not been clearly elucidated. The expression and activity of PKG II in human gastric cancer cell lines have been observed to be significantly lower than those of normal gastric mucosal cells, and the increase of PKG II activity may inhibit the proliferation of gastric cancer cell lines (9,10). Further study in our laboratory revealed that PKG II was able to inhibit EGF-induced proliferation and migration, and inhibit MAPK-mediated signal transduction in gastric cancer cells. Notably, the inhibition was associated with the prevention of EGF-induced phosphorylation/activation of EGFR (11,12). As the activation of EGFR also initiates other signal transduction pathways, including the PI3K/Akt- and JAK/STAT-mediated pathways (5,13,14), further investigation into whether PKG II has an inhibitory effect on these signal pathways is required. The present study was designed to elucidate the possible inhibition of PKG II on the EGF-initiated PI3K/Akt-mediated signal transduction pathway.

## Materials and methods

### Cell line and reagents

The AGS human gastric cancer cell line was provided by the Institute of Cell Biology (Shanghai, China). Adenoviral vectors encoding β-galactosidase (pAd-LacZ) and PKG II (pAd-PKG II) were provided by the University of California (San Diego, CA, USA). Dulbecco’s modified Eagle’s medium (DMEM) and fetal bovine serum (FBS) were obtained from Gibco (Grand Island, NY, USA). The antibody against PKG II (rabbit anti-human) was from Abgent Biotechnology (San Diego, CA, USA). The horseradish peroxidase (HRP)-conjugated antibody against β-actin (moust anti-human) and the antibody against Bcl-2 (mouse anti-human) were obtained from Santa Cruz (Santa Cruz, CA, USA). The antibody against p-EGFR (Tyr1173; rabbit anti-human) was purchased from Cell Signaling (Danvers, MA, USA). The antibodies against p-PI3K, p-Akt, p-mTOR, NF-κB, Bax, DNA fragment factor (DFF), caspase-9 and LaminB1 (rabbit anti-human) were purchased from Bioworld Technology (St. Louis Park, MN, USA). HRP-conjugated secondary antibodies (goat anti-mouse and goat anti-rabbit) were obtained from Jackson Immuno Research Laboratories (West Grove, PA, USA). The cellular permeable cGMP analog 8-pCPT-cGMP was purchased from Calbiochem (San Diego, CA, USA) and EGF was obtained from Sigma (St. Louis, MO, USA). The electrochemiluminescence (ECL) reagents were purchased from Millipore (Billerica, MA, USA). The terminal deoxynucleotidyl transferase-mediated dUTP nick end-labeling (TUNEL) In Situ Cell Death Detection kit was purchased from Roche Diagnostics (Mannheim, Germany), while the Nuclear and Cytoplasmic Extract kit was purchased from Kangcheng Bio-tech (Hangzhou, China). All other reagents used were of analytical grade.

### Cell culture and treatment

The AGS cells were cultured in DMEM, supplied with 10% FBS and maintained at 37ºC in a humidified incubator with 95% air and 5% CO_2_. One day prior to the infection, the cells were planted into six-well plates. When the cells were 70–80% confluent, they were infected with Ad-LacZ or Ad-PKG II (with a multiplicity of infection of 100%) or mock infected. At 24 h post-infection, the medium was replaced with serum-free medium and the culture was continued for 12 h. The infected cells were incubated with 100 or 250 μM 8-pCPT-cGMP for 1 h and incubated with 100 ng/ml EGF. The EGF incubation time was 5 min to observe EGFR phosphorylation and 12 h to observe the change in protein expression.

### Preparation of cell extracts and nuclear protein

The differentially treated cells were harvested at various times by aspiration of the media and direct addition of heated 2X SDS sample buffer. The cell lysate was scraped and transferred to tubes, heated for 5 min at 100ºC and stored at −20ºC. The nuclear extracts were prepared according to the instructions of the manufacturer of the Nuclear and Cytoplasmic Extract kit. Briefly, the cells were harvested into tubes containing PBS and centrifuged at 500 × g for 5 min. Ice-cold cytoplasmic extraction reagent (CER) I buffer was added to the cell pellet and fully resuspended by vortexing. The tubes were then incubated on ice for 10 min followed by an addition of ice-cold CER II buffer, vortexed and incubated on ice for 1 min. The samples were centrifuged for 5 min in a microcentrifuge (~16,000 × g). The supernatants (cytoplasmic extracts) were immediately transferred to a clean pre-chilled tube. The insoluble fractions (pellets) were resuspended in ice-cold NER buffer. The samples were repeatedly vortexed for 15 sec every 10 min (at 4ºC) for a total of at least 40 min and microcentrifuged (~16,000 × g) for 10 min. The supernatant fractions (nuclear extracts) were immediately transferred to pre-chilled tubes and stored at −20ºC until use.

### Western blotting

The proteins were separated by SDS-PAGE (8–12%) gel according to their molecular size and transferred onto a PVDF membrane. The blots were blocked using 5% (w/v) non-fat milk in TBS-T for 1 h at room temperature, and then incubated at 4ºC overnight with the primary antibody. Subsequently, the blots were incubated with the secondary antibody at room temperature for 1 h. The signal was visualized using ECL detection reagents.

### Detection of apoptosis using the TUNEL method

TUNEL was performed using the In Situ Cell Death Detection kit, according to the manufacturer’s instructions. Briefly, the cells that were grown on 24-well plates were fixed and the endogenous peroxidase activity was quenched using 2% H_2_O_2_ for 5 min. The cells were incubated with a reaction buffer containing terminal deoxynucleotidyl transferase, 1 mM Mn^2+^ and fluorescein-labeled dUTP in a humid atmosphere at 37ºC for 60 min. The cells were washed with PBS and incubated with antifluorescein antibody conjugated with HRP for 30 min. Subsequent to being rinsed with PBS, the cells were immersed in DAB solution. Under a microscope, the cells that exhibited brown nuclear staining were considered to be apoptotic. For each well, five fields (magnification, ×200) were randomly selected, and the number of positive cells and the total number of cells in each field were counted. The average ratio (positive cell number/total cell number) of the apoptotic cells was taken as the value for one experiment. The assay was repeated three times.

### Statistical analysis

Data are expressed as the mean ± standard deviation. Statistical analysis was performed using a two-tailed ANOVA with SPSS statistical software (SPSS, Inc., Chicago, IL, USA). P<0.05 was considered to indicate a statistically significant difference.

## Results

### PKG II inhibits EGF-induced Tyrosine 1173 (Tyr1173) phosphorylation/activation of EGFR

**Figure 1:**
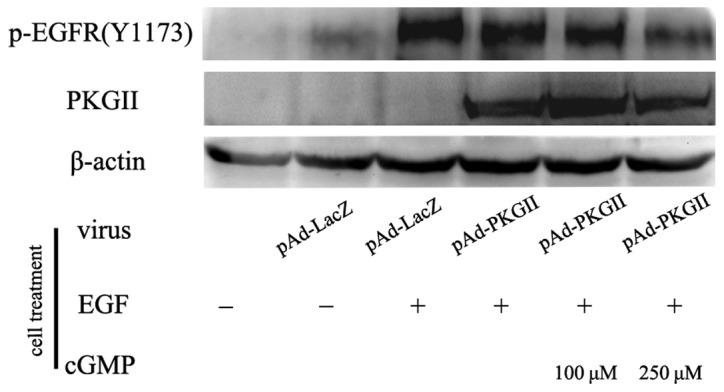
PKG II inhibits EGF-induced Y1173 phosphorylation of EGFR. The AGS cells were infected with Ad-LacZ or Ad-PKG II for 24 h and serum starved overnight The cells in the Ad-LacZ+EGF and Ad-PKG II+EGF groups were incubated with 100 ng/ml EGF for 5 min. The cells in the Ad-PKG II+cGMP+EGF groups were incubated with 8-pCPT-cGMP for 1 h and then with 100 ng/ml EGF for 5 min. The cells were harvested and lysed as described in Materials and methods and the cell lysate was subjected to western blotting. The results revealed that the infection with Ad-PKG II caused a marked increase in PKG II expression. EGF treatment induced an increase in Y1173 phosphorylation of EGFR. Infection with Ad-PKG II+cGMP efficiently inhibited the EGF-induced phosphorylation of EGFR. PKG II, cGMP-dependent protein kinase Type II; EGFR, epidermal growth factor receptor.

When EGF binds with EGFR, Tyr1173 is one of the autophosphorylation sites that is located on the receptor. The phosphorylation of this site is associated with PI3K/Akt-mediated signaling. In the present study, the inhibitory effect of PKG II on the Tyr1173 phosphorylation of EGFR was investigated in the differentially treated AGS cells, using western blotting. The results revealed that in the Ad-LacZ-infected cells, there was a marked increase in the phosphorylated Tyr1173 residues of EGFR when the cells were incubated with 100 ng/ml EGF for 5 min. In the cells that were infected with Ad-PKG II, stimulated with cGMP and incubated with 100 ng/ml EGF for 5 min, the phosphorylation was markedly decreased (Fig. 1). This indicated that PKG II was able to inhibit Tyr1173 phosphorylation/activation of EGFR caused by EGF.

### PKG II inhibits EGF-induced phosphorylation/activation of PI3K

**Figure 2:**
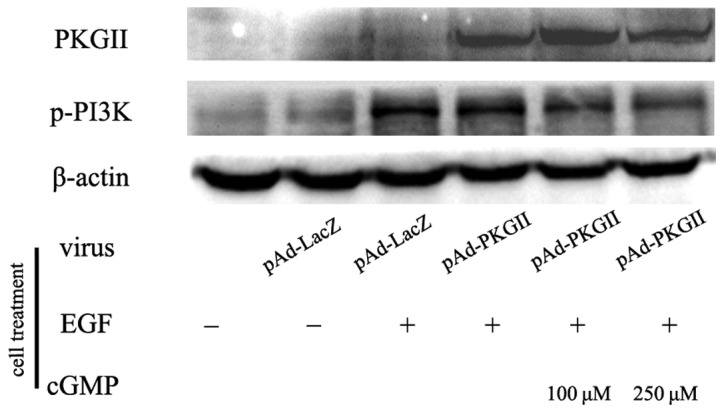
PI3K phosphorylation/activation is induced by EGF and inhibited by PKG II. The AGS cells were treated as in Fig. 1 and western blotting with an antibody against p-PI3K p85 (Tyr458) was performed to analyze the effect of EGF and PKG II on the phosphorylation/activation of PI3K. The results revealed that in the EGF-treated cells, the phosphorylation level of PI3K p85 (Tyr458) increased. Infection with Ad-PKG II+cGMP efficiently inhibited the EGF-induced phosphorylation of PI3K p85 (Tyr458). PI3K, phosphatidylinositol-3-kinase; EGF, epidermal growth factor; PKG II, cGMP-dependent protein kinase Type II.

Phosphorylated EGFR is able to recruit the PI3K regulatory subunit, p85α, to its Tyr1173 site and activate the lipid kinase. During the activation processes, Tyr458 on p85α is phosphorylated. Western blotting with an antibody against p-PI3K p85 (Tyr458) was used to detect the phosphorylation of p85α. The AGS cells were treated as previously described and the western blotting results revealed that EGF treatment (100 ng/ml for 5 min) increased the phosphorylation of PI3K p85α. In the cells that were infected with Ad-PKG II, stimulated with cGMP and treated with EGF, the phosphorylation level of PI3K p85α was markedly lower than that of the cells that were infected with Ad-LacZ or treated with EGF alone (Fig. 2). These results revealed that PKG II inhibited the EGF-induced activation of PI3K.

### PKG II inhibits EGF-induced phosphorylation/activation of Akt

**Figure 3:**
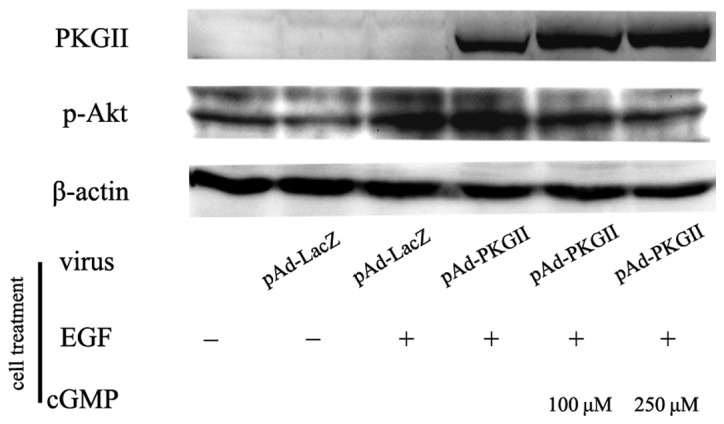
PKG II inhibits EGF-induced phosphorylation/activation of Akt. The AGS cells were treated as in Fig. 1 and western blotting with an anti p-Akt (Thr308) antibody was performed to analyze the effect of EGF and PKG II on the phosphorylation/activation of Akt. The results revealed that in the EGF-treated cells, the phosphorylation level of Akt (Thr308) increased. Infection with Ad-PKG II+cGMP efficiently inhibited the EGF-induced phosphorylation of Akt (Thr308). PKG II, cGMP-dependent protein kinase Type II; EGF, epidermal growth factor.

Akt is a crucial signal transduction component in the PI3K-mediated pathway. The activation of Akt is dependent on a dual regulatory mechanism that requires its translocation to the plasma membrane and dual phosphorylation on Thr308 and Ser473. In the present study, the AGS cells were treated as previously described and western blotting with an antibody against p-Akt (Thr308) was used to detect the phosphorylation/activation of Akt by EGF. The results revealed that EGF treatment induced a notable increase in Thr308 phosphorylation on Akt, and the increase of PKG II activity effectively inhibited the EGF-induced Akt phosphorylation/activation of Akt (Fig. 3).

### EGF-induced phosphorylation/activation of mTOR is inhibited by activated PKG II

**Figure 4:**
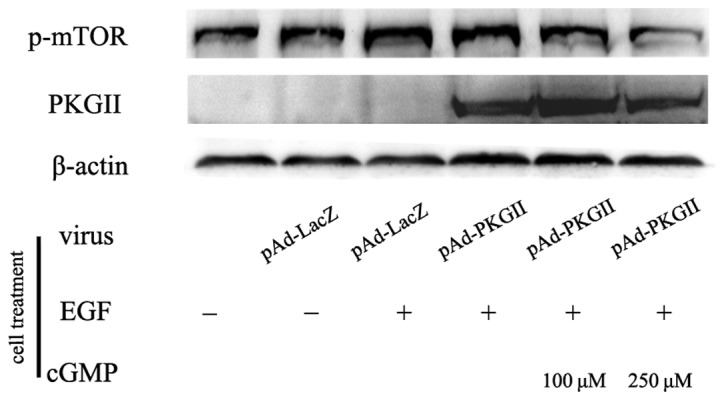
PKG II prevents EGF-induced phosphorylation/activation of mTOR. The AGS cells were treated as in Fig. 1 and western blotting with an anti p-mTOR (Ser2448) antibody was performed to analyze the effect of EGF and PKG II on the phosphorylation/activation of mTOR. The results revealed that in the EGF-treated cells, the phosphorylation level of mTOR (Ser2448) increased. Infection with Ad-PKG II+cGMP efficiently inhibited the EGF-induced phosphorylation of mTOR (Ser2448). PKG II, cGMP-dependent protein kinase Type II; EGF, epidermal growth factor.

mTOR has been shown to be a direct substrate for AKT. The proposed AKT phosphorylation site (Ser2448) in mTOR is located within a C-terminal regulatory region. In the present study, the AGS cells were treated as in Fig. 1 and western blotting with an anti p-mTOR (Ser2448) antibody was used to detect the phosphorylation of mTOR in the various cell groups. The results showed that EGF treatment (100 ng/ml, 5 min) caused a marked increase of Ser2448 phosphorylation of mTOR, and the increase in PKG II activity through infecting the cells with pAd-PKG II and stimulating the cells with 8-pCPT-cGMP efficiently inhibited the EGF-induced phosphorylation/activation of mTOR (Fig. 4).

### PKG II inhibits EGF-induced activation of NF-κB

**Figure 5:**
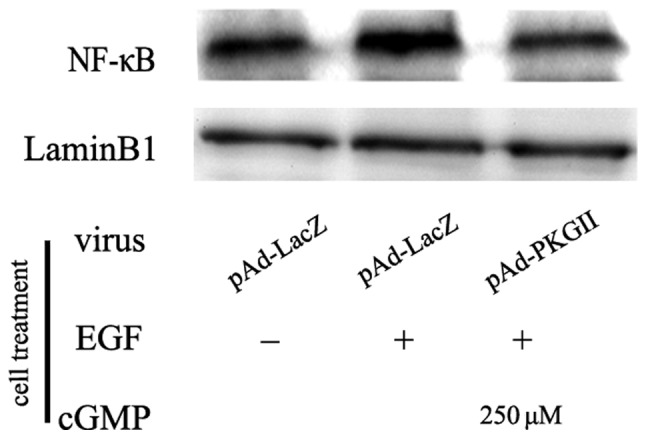
PKG II inhibits EGF-induced activation of NF-κB. The AGS cells were infected with Ad-LacZ or Ad-PKG II for 24 h and serum starved overnight. The cells in the LacZ+EGF group were incubated with 100 ng/ml EGF for 12 h. The cells in the Ad-PKG II+cGMP+EGF group were treated with 8-pCPT-cGMP for 1 h and 100 ng/ml EGF was added to the medium and the incubation was continued for 12 h. The cells were harvested and the nuclear extract was prepared according to the instructions of the manufacturer of the Nuclear and Cytoplasmic Extract kit. The nuclear extract was subjected to western blotting with an antibody against p65 NF-κB. The results revealed that in the EGF-treated cells, the nuclear location of NF-κB increased. Infection with Ad-PKG II+cGMP efficiently inhibited the EGF-induced increase in the nuclear localization of NF-κB. These results indicated that PKG II inhibited the EGF-induced activation of NF-κB. PKG II, cGMP-dependent protein kinase Type II; EGF, epidermal growth factor.

Transcription factor NF-κB is a dimer formed by a group of subunits, including Rel (cRel), p65 (RelA, NF-κB3), RelB, p50 (NF-κB1) and p52 (NF-κB2). The most common NF-κB is the heterogeneous dimmer formed by p65 and p50. When NF-κB is activated, the dimer translocates into the nucleus, binds with a corresponding promoter and initiates gene transcription. In the present study, western blotting with an anti-p65 (RelA) antibody was used to detect the nuclear translocation of NF-κB. The results revealed that the EGF treatment caused an increase in the nuclear localization of p65 NF-κB. PKG II activity prevented the translocation, indicating that PKG II is able to inhibit the EGF-induced nuclear translocation/activation of NF-κB (Fig. 5).

### PKG II reverses EGF-induced expression of apoptosis regulator proteins

**Figure 6:**
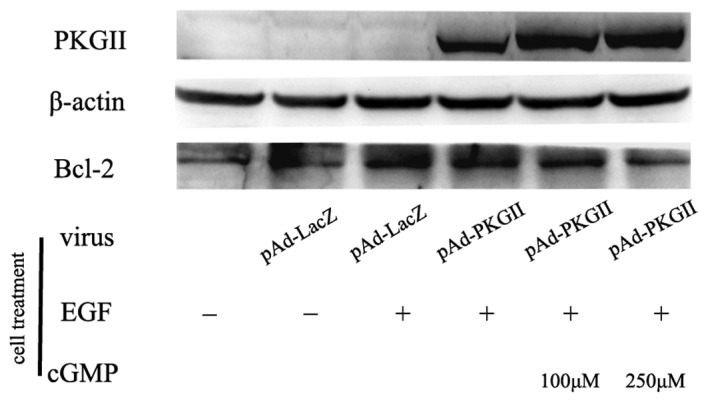
PKG II inhibits EGF-induced expression of Bcl-2. The AGS cells were treated as in Fig. 5 and harvested and lysed as described in Materials and methods. The cell lysate was subjected to western blotting with an anti Bcl-2 antibody. The results revealed that in the EGF-treated cells, the expression of Bcl-2 increased, and a higher expression and activity of PKG II prevented the EGF-induced increase of Bcl-2 expression. PKG II, cGMP-dependent protein kinase Type II; EGF, epidermal growth factor.

**Figure 7:**
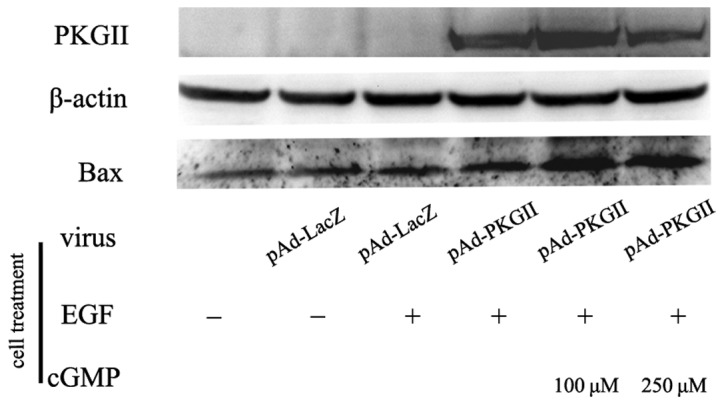
PKG II increases the expression of Bax. The AGS cells were treated as in Fig. 5 and harvested and lysed as described in Materials and methods. The cell lysate was subjected to western blotting with an anti-Bax antibody. The results revealed that in the EGF-treated cells, the expression of Bax did not markedly change. However, higher expression and activity of PKG II caused a marked increase in the expression of Bax. PKG II, cGMP-dependent protein kinase Type II; EGF, epidermal growth factor.

Bcl-2 is an anti-apoptotic protein and a member of the Bcl-2 family of the apoptosis regulator proteins. In the present study, the expression of Bcl-2 in the EGF-treated AGS cells was detected by western blotting. The results revealed that EGF treatment (100 ng/ml, 12 h) caused an increase in the expression of Bcl-2. Pre-infection with Ad-PKG II and treatment with 8-pCPT-cGMP reversed the effect of EGF, causing a marked decrease in Bcl-2 expression (Fig. 6). In contrast, EGF treatment did not cause a marked change in the expression of the pro-apoptotic protein, Bax, which, if activated, leads to the activation of caspases and apoptosis. However, an increase of PKG II activity caused a clear increase in Bax expression (Fig. 7).

### PKG II stimulates the activation of caspase-9

**Figure 8:**
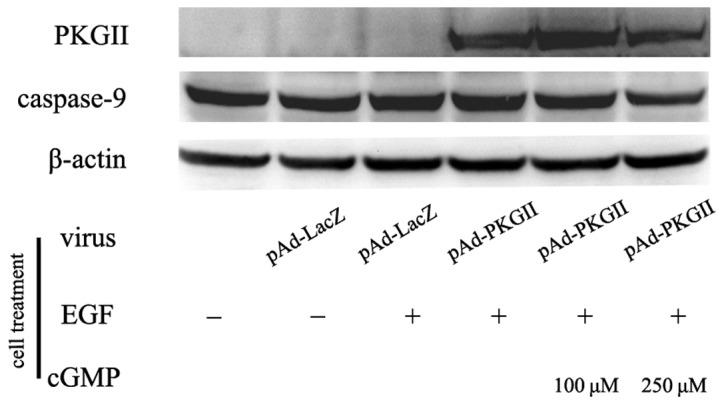
PKG II inhibits the activation of caspase-9. The AGS cells were treated as in Fig. 5 and western blotting with an anti caspase-9 antibody was performed to analyze the effect of EGF and PKG II on the breakdown (activation) of caspase-9. The results revealed that in the EGF-treated cells, the activation of caspase-9 did not change. A higher expression and activity of PKG II markedly increased the activation of caspase-9. PKG II, cGMP-dependent protein kinase Type II; EGF, epidermal growth factor.

Caspases are essential in the regulation of cell apoptosis and have been termed ‘executioner’ proteins. In the present study, the changes in the protein levels of caspase-3 and caspase-9 were detected by western blotting. The results revealed that EGF treatment had no effect on the levels of caspase-3 (not shown) and caspase-9. An increase in PKG II activity had no effect on caspase-3 protein levels (not shown). However, PKG II did cause a marked decrease in caspase-9. This suggests that PKG II had a stimulating effect on the activation of caspase-9 (Fig. 8).

### PKG II increases the activity of DFF

**Figure 9:**
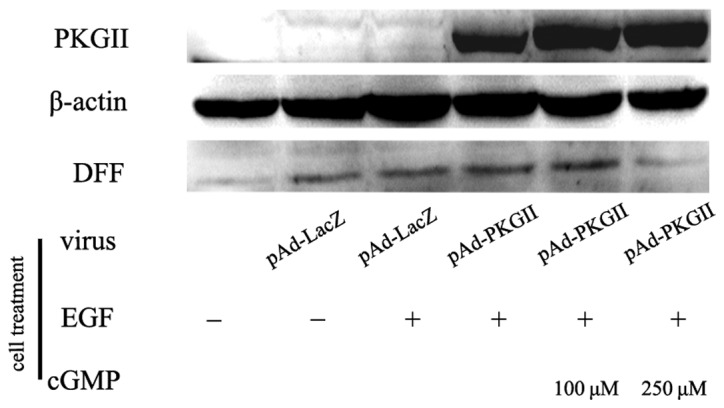
PKG II inhibits the activation of DFF. The AGS cells were treated as in Fig. 5 and western blotting with an anti DFF antibody was performed to analyze the effect of EGF and PKG II on the breakdown (activation) of DFF. The results revealed that in the EGF-treated cells, the activation of DFF did not change. A higher expression and activity of PKG II markedly increased the activation of DFF. PKG II, cGMP-dependent protein kinase Type II; EGF, epidermal growth factor; DFF, DNA fragment factor.

DFF is the component located at the end of apoptotic pathway. Apoptotic signaling is able to stimulate DFF to break into an active form, cause DNA fragmentation and initiate apoptosis. Western blotting was used to detect the change in the protein levels of DFF during EGF and PKG II/cGMP treatment. The results revealed that EGF treatment had no effect on the protein levels of DFF. However, an increase in PKG II activity caused a decrease in DFF protein levels, indicating that PKG II activated DFF (Fig. 9). This suggests that PKG II is able to increase the activity of DFF, cause DNA fragmentation and increase the apoptosis of AGS cells.

### PKG II reverses the anti-apoptotic effect of EGF

**Figure 10:**
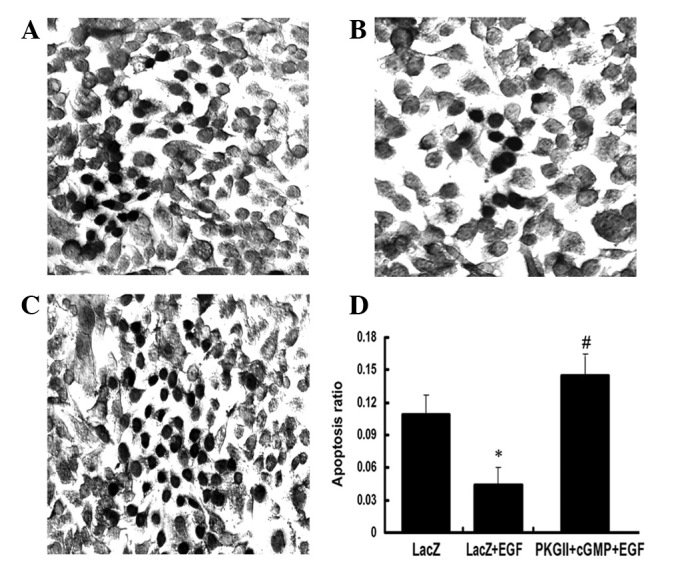
PKG II reverses the anti-apoptotic effect of EGF. The AGS cells were treated as in Fig. 5 and TUNEL staining was performed according to the manufacturer’s instructions of the In Situ Cell Death Detection kit. The cells with dark brown nuclear staining were considered to be apoptotic and were counted as described in Materials and methods. (A–C) Representatives of the staining. (A) The cells were infected with Ad-LacZ only. (B) The cells were infected with Ad-LacZ and incubated with 100 ng/ml EGF for 12 h. (C) The cells were infected with Ad-PKG II, incubated with 250 μM 8-pCPT-cGMP for 1 h and incubated with 100 ng/ml EGF for 12 h. (D) The average ratio of apoptotic cells per field (magnification, ×200) of each group (^*^P<0.05, compared with the LacZ group and ^#^P<0.05, compared with the LacZ+EGF group). The results revealed that EGF treatment decreased the apoptosis of the AGS cells, while pre-infecting with Ad-PKG II and pre-incubating with 250 μM 8-pCPT-cGMP efficiently reversed the effect of EGF. PKG II, cGMP-dependent protein kinase Type II; EGF, epidermal growth factor; TUNEL, terminal deoxynucleotidyl transferase-mediated dUTP nick end-labeling.

The TUNEL method was used to detect the apoptosis of the differentially treated AGS cells. In the EGF treatment group (100 ng/ml, 12 h) the ratio of apoptotic cells markedly decreased. Ad-PKG II infection and incubating with 8-pCPT-cGMP efficiently reversed the anti-apoptotic effect of EGF and caused a significant increase in the apoptosis of the AGS cells (Fig. 10).

## Discussion

PKG II has long been implicated in several physiological functions, including intestinal secretion, bone growth, learning and memory (15). However, certain new functions of this kinase have been reported, including the regulation of epithelial sodium channels and mechanotransduction of osteoblasts (16–18). A significant observation in studies with regard to PKG II is that this kinase has a role in regulating the proliferation and apoptosis of cells and is potentially associated with tumorigenesis. Data have shown PKG II to inhibit the proliferation of human prostate and neuroglioma cells, and induce the apoptosis of human prostate and breast cancer cells (19–21). The present study confirmed the inhibitory effect of PKG II on the proliferation and migration of cancer cells, and the associated mechanisms are currently under extensive investigation. A significant finding is that PKG II is able to inhibit EGF-induced activation of EGFR, which is the starting event of the signal transduction of EGF/EGFR mediated pathways (11,12).

Activation of EGFR initiates the signal transduction of several pathways, including the MAPK-, PI3K/Akt-, PLCγ1/IP3/DAG- and JAK1/STAT-mediated pathways. The signal transductions of these pathways are associated with the proliferation, apoptosis and motility/migration of the cells (5,14,22). Therefore, further study is required with regard to whether PKG II also inhibits the signal transduction of other pathways besides those that are mediated by MAPK, and whether it affects cellular activities other than proliferation and migration. The present study confirmed that in the gastric cancer AGS cell line, EGF caused Tyr1173 phosphorylation of EGFR and the consequent activation of the key components of the PI3K/Akt-mediated pathway, including PI3K, Akt, mTOR and NF-κB. PKG II was also identified to suppress the whole process of EGF-induced PI3K/Akt-mediated signal transduction, including the activation of EGFR and each key signaling component of the pathway.

The PI3K/Akt-mediated signal pathway is significant in regulating apoptosis (23). In numerous cancer tissues and cells, this pathway is overactive, reducing apoptosis and allowing proliferation. The present study identified that EGF treatment significantly reduced the apoptosis of the AGS cells. This effect was prevented by a PI3K inhibitor (data not shown), indicating that EGF was not able to reduce apoptosis of the gastric cancer cells through the PI3K/Akt-mediated pathway. PKG II was also able to efficiently reverse the anti-apoptotic effect of EGF. This suggests that PKG II not only inhibits the proliferation but also induces the apoptosis of gastric cancer cells, and the two effects are associated with the blockage of EGFR activation by this kinase.

EGFR is closely associated with tumorigenesis. The overexpression and mutations of EGFR are identified in the majority of cancer types (24). *In vitro* experiments have confirmed that blocking EGFR activation inhibited the proliferation of certain types of tumor cells (25). Furthermore, a clinical study has shown that cancer patients with an overexpression of EGFR usually have a poor prognosis (26). Therefore, EGFR is a potential cancer therapy target and methods of inhibiting EGFR activity and associated signal transduction have been intensively studied, including specific antibodies against EGFR and inhibitors of EGFR (27). The present data have shown that PKG II inhibits EGF/EGFR-induced proliferation and migration and associated signal transduction of MAPK-mediated pathways of cancer cells. The present study revealed that in gastric cancer cells, PKG II is able to inhibit the EGF/EGFR-induced signal transduction of the PI3K/Akt-mediated pathway and reverse the anti-apoptotic effects of EGF/EGFR. This strongly suggests that PKG II is a cancer suppression factor and may influence the strategy of cancer therapy.
